# *N*-Glycosylation Regulates the Trafficking and Surface Mobility of GluN3A-Containing NMDA Receptors

**DOI:** 10.3389/fnmol.2018.00188

**Published:** 2018-06-04

**Authors:** Kristyna Skrenkova, Sanghyeon Lee, Katarina Lichnerova, Martina Kaniakova, Hana Hansikova, Martin Zapotocky, Young Ho Suh, Martin Horak

**Affiliations:** ^1^Department of Cellular Neurophysiology, Institute of Physiology of the Czech Academy of Sciences, Prague, Czechia; ^2^Department of Neurochemistry, Institute of Experimental Medicine of the Czech Academy of Sciences, Prague, Czechia; ^3^Department of Physiology, Faculty of Science, Charles University in Prague, Prague, Czechia; ^4^Department of Biomedical Sciences, Neuroscience Research Institute, Seoul National University College of Medicine, Seoul, South Korea; ^5^Department of Pediatrics and Adolescent Medicine, First Faculty of Medicine, Charles University in Prague and General University Hospital in Prague, Prague, Czechia; ^6^Department of Computational Neuroscience, Institute of Physiology of the Czech Academy of Sciences, Prague, Czechia

**Keywords:** glutamate receptor, glycan, endoplasmic reticulum, excitatory synapse, ion channel, mammalian neuron

## Abstract

*N*-methyl-D-aspartate receptors (NMDARs) play critical roles in both excitatory neurotransmission and synaptic plasticity. NMDARs containing the nonconventional GluN3A subunit have different functional properties compared to receptors comprised of GluN1/GluN2 subunits. Previous studies showed that GluN1/GluN2 receptors are regulated by *N*-glycosylation; however, limited information is available regarding the role of *N*-glycosylation in GluN3A-containing NMDARs. Using a combination of microscopy, biochemistry, and electrophysiology in mammalian cell lines and rat hippocampal neurons, we found that two asparagine residues (N203 and N368) in the GluN1 subunit and three asparagine residues (N145, N264 and N275) in the GluN3A subunit are required for surface delivery of GluN3A-containing NMDARs. Furthermore, deglycosylation and lectin-based analysis revealed that GluN3A subunits contain extensively modified *N*-glycan structures, including hybrid/complex forms of *N*-glycans. We also found (either using a panel of inhibitors or by studying human fibroblasts derived from patients with a congenital disorder of glycosylation) that *N*-glycan remodeling is not required for the surface delivery of GluN3A-containing NMDARs. Finally, we found that the surface mobility of GluN3A-containing NMDARs in hippocampal neurons is increased following incubation with 1-deoxymannojirimycin (DMM, an inhibitor of the formation of the hybrid/complex forms of *N*-glycans) and decreased in the presence of specific lectins. These findings provide new insight regarding the mechanisms by which neurons can regulate NMDAR trafficking and function.

## Introduction

*N*-methyl-D-aspartate receptors (NMDARs), a subclass of glutamate receptors, are essential for excitatory neurotransmission and synaptic plasticity in the mammalian central nervous system (CNS; Traynelis et al., 2010; Horak et al., 2014). NMDARs are comprised of GluN1 (with eight splice variants), GluN2 (GluN2A through GluN2D), and GluN3 (GluN3A and GluN3B) subunits, all of which have a similar membrane topology. The GluN3A subunit is a recently discovered subunit that co-assembles with GluN1 and GluN2 subunits to form small-conductance GluN1/GluN2/GluN3 receptors; alternatively, GluN3A can co-assemble exclusively with the GluN1 subunit, forming the glycine-activated GluN1/GluN3 receptor (Traynelis et al., 2010; Pachernegg et al., 2012; Kehoe et al., 2013). Several studies showed that the GluN3A subunit is expressed robustly in the rodent brain during early postnatal development; moreover, this subunit plays a role in inhibiting the maturation of excitatory glutamatergic synapses during neural development (Wong et al., 2002; Fiuza et al., 2013; Yuan and Bellone, 2013).

Although many studies have examined the molecular mechanisms that regulate the surface expression and surface mobility of NMDARs, these studies focused largely on NMDAR subtypes containing GluN1/GluN2 subunits (Traynelis et al., 2010; Sanz-Clemente et al., 2012). With respect to posttranslational modifications of GluN3A-containing NMDARs, both tyrosine phosphorylation of the endocytic motif within the C-terminus and interaction between the GluN3A subunit and the endocytic adaptor protein kinase C and casein kinase substrate in neurons 1 (PACSIN1) regulate the surface expression of these NMDARs (Pérez-Otaño et al., 2006; Chowdhury et al., 2013). GluN1/GluN2 receptors have been found to be extensively *N*-glycosylated in both heterologous expression systems (Chazot et al., 1995; Everts et al., 1997) and native preparations (Huh and Wenthold, 1999; Kaniakova et al., 2016); moreover, we recently reported that two conventional *N*-glycosylation sites in the GluN1 subunit are required for the release of GluN1/GluN2 receptors from the endoplasmic reticulum (ER; Lichnerova et al., 2015); however, the role of *N*-glycosylation in the trafficking and/or surface mobility of NMDARs containing the nonconventional GluN3A subunit has not been examined.

Here, using a combination of microscopy, biochemistry and electrophysiology in mammalian cell lines and primary hippocampal neurons, we examined the role that *N*-glycosylation plays in the trafficking and mobility of GluN3A-containing NMDARs. We found that two previously identified *N-glycosylation* sites (N203 and N368) in the GluN1 subunit, as well as three *N*-glycosylation sites in the N-terminal domain of the GluN3A subunit, are required for surface delivery of GluN3A-containing NMDARs. We also performed deglycosylation and lectin-based analyses of recombinant and endogenous GluN3A subunits, revealing that this subunit contains a wide diversity of *N*-glycans, including hybrid/complex types. In addition, we found that altering the early steps in the *N*-glycosylation pathway reduces the surface delivery of GluN3A-containing NMDARs, whereas preventing the subsequent remodeling of *N*-glycans has no effect. Finally, we found that 1-deoxymannojirimycin (DMM), which inhibits the formation of hybrid/complex forms of *N-glycans*, increases the surface mobility of GluN3A-containing NMDARs, whereas the lectins conA, WGA, and AAL slow surface mobility. Taken together, these results indicate that *N*-glycosylation regulates the trafficking and surface mobility of GluN3A-containing NMDARs in mammalian neurons.

## Materials and Methods

### Mammalian Expression Vectors

The untagged GluN1–4a expression vector was obtained from Dr. Robert J. Wenthold (NIDCD, NIH), and the GFP-GluN3A vector was a kind gift from Pérez-Otaño et al. (2001). Point mutations were introduced using the Quick-Change site-directed mutagenesis kit (Agilent Technologies), and the entire GluN-coding region was confirmed by sequencing. The following multiple-site mutant versions of the GluN constructs were generated: GluN1-N61Q-N203Q-N239Q- N276Q-N300Q-N350Q = GluN1–1/2N→Q; GluN1-N368Q-N440Q-N471Q-N491Q-N674Q-N771Q = GluN1–2/2N→Q; GluN1-N61Q-N203Q-N239Q = GluN1–1/4N→Q; GluN1-N276Q-N300Q-N350Q = GluN1–2/4N→Q; GluN1-N368Q-N440Q-N471Q = GluN1–3/4N→Q; GluN1-N491Q-N674Q-N771Q = GluN1–4/4N→Q; GluN3A-N145Q-N264Q-N275Q-N285Q-N296Q-N300Q-N320Q-N426Q-N439Q-N549Q-N565Q-N886Q = GluN3A-12N→12Q; GluN3A-N145Q-N264Q-N275Q-N285Q-N296Q-N300Q = GluN3A-1/2N→Q; GluN3A-N320Q-N426Q-N439Q-N549Q-N565Q-N886Q = GluN3A-2/2N→Q; GluN3A-N145Q-N264Q-N275Q = GluN3A-1/4N→Q; GluN3A-N285Q-N296Q-N300Q = GluN3A-2/4N→Q; GluN3A-N320Q-N426Q-N439Q = GluN3A-3/4N→Q; GluN3A-N549Q-N565Q-N886Q = GluN3A-4/4N→Q (Supplementary Figures S1, S2).

### Mammalian Cell Culture and Transfection

Heterologous African green monkey kidney fibroblast (COS-7) cells and human embryonic kidney 293 (HEK293) cells were cultured in Opti-MEM I media containing 5% (v/v) fetal bovine serum (FBS; Thermo Fisher Scientific). Human fibroblasts were grown in DMEM media (FG0445; Baria) containing 10% FBS and 1% (w/v) gentamycin (Thermo Fisher Scientific). All animal experiments were performed in accordance with institutional ethics guidelines and regulations for ensuring animal welfare. The experiments involving human fibroblasts were performed in accordance with the Declaration of Helsinki of the World Medical Association and was approved by each institute’s respective Medical Ethics Committee. Informed and written consent was obtained from the parents of the patients who provided fibroblasts. All cell lines were transfected with DNA vectors using Lipofectamine 2000 (Thermo Fisher Scientific; Kaniakova et al., 2012a). After transfection, the HEK293 cells used for electrophysiology were dissociated using trypsin. The cells used for microscopy and biochemistry were cultured without the trypsinization step. Experiments were performed within 24–48 h of transfection.

### Preparation and Transfection of Primary Hippocampal Neurons

All animal experiments were conducted in accordance with the guidelines of the European Union directive 2010/63/EU and approved by the Animal Care and Use Committee of the Institute of Physiology of the Czech Academy of Science (CAS). The Institute of Physiology CAS possesses the National Institutes of Health Statement of Compliance with Standards for Humane Care and Use of Laboratory Animals. Primary cultures of hippocampal neurons were prepared from embryonic day 18 Wistar rats (Lichnerova et al., 2015). In brief, the hippocampi were collected in cold Hank’s Balanced Salt Solution containing 10 mM HEPES (pH 7.4), and then incubated for 20 min at 37°C in dissection media containing 0.1 mg/ml DNase I and 0.05% trypsin (Merck). The cells were then washed, dissociated by trituration through a fire-polished glass pipette, and resuspended in plating medium consisting of serum-free Neurobasal media with B-27 supplement and L-glutamine (Thermo Fisher Scientific). The cells were grown at a density of approximately 2 × 10^4^ cells per cm^2^ on dishes coated with poly-L-lysine (Sigma). The neurons were fed every 2–3 days with plating media and transfected using Lipofectamine 2000.

### Electrophysiology

Whole-cell patch-clamp recordings of HEK293 cells were performed using an Axopatch 200B amplifier (Molecular Devices). The extracellular recording solution contained (in mM): 160 NaCl, 2.5 KCl, 10 HEPES, 10 glucose, 0.2 ethylenediaminetetraacetic acid (EDTA), and 0.7 CaCl_2_ (pH adjusted to 7.3 with NaOH). The internal pipette solution contained (in mM): 125 gluconic acid, 15 CsCl, 5 BAPTA, 10 HEPES, 3 MgCl_2_, 0.5 CaCl_2_, and 2 ATP-Mg salt (pH adjusted to 7.2 with CsOH). Glass patch pipettes (3–6 MΩ resistance when filled with internal solution) were prepared using a model P-97 micropipette puller (Sutter Instrument Co.). A microprocessor-controlled multi-barrel rapid perfusion system with a time constant for solution exchange around the cell of 10–20 ms was used to apply a solution containing glycine (Kaniakova et al., 2016). All electrophysiology experiments were performed at room temperature.

### Immunofluorescence Microscopy

Surface NMDARs were labeled as described previously (Kaniakova et al., 2012a; Lichnerova et al., 2015). In brief, the cells were washed in phosphate-buffered saline (PBS), and then incubated in blocking solution containing PBS and 10% (v/v) normal goat serum. The cells were then incubated for 30 min with the primary antibody diluted in blocking solution. After washing, the cells were incubated for 30 min with a secondary antibody conjugated with a fluorescent dye and diluted in blocking solution. The cells were washed and then fixed in 4% (w/v) paraformaldehyde (PFA) in PBS for 20 min, and then mounted using ProLong Antifade reagent (Thermo Fisher Scientific). For co-localization studies and for labeling intracellular GluN3A subunits, the cells were washed in PBS, fixed with 4% PFA in PBS for 20 min, permeabilized for 5 min with 0.25% (w/v) Triton X-100 (TX-100) in PBS, and then blocked for 1 h with blocking solution containing 0.1% TX-100. The cells were then incubated with the primary antibody for 1 h, washed, and incubated with the secondary antibody for 30 min. Images were acquired at room temperature using a fluorescence microscope (Olympus Scan) with a 60×/1.35 oil immersion objective or a confocal scanning microscope (Leica TCS SPE) fitted with solid-state lasers and a 63×/1.30 oil immersion apochromat objective. The images were analyzed using ImageJ software (NIH). The intensity of the surface and total GFP signals in the COS-7 cells and fibroblasts were analyzed on whole-cell areas (Kaniakova et al., 2012b). For hippocampal neurons, the intensity of the surface and total GFP signals was analyzed in 10-μm long segments of secondary and tertiary dendrites (Lichnerova et al., 2015). The spine/dendrite fluorescence intensity ratio was measured across the dendrite and spine and analyzed using a line plot. The maximum fluorescence intensity of each dendrite and adjacent spine was used to calculate the spine/dendrite ratio as described previously (Gerges et al., 2004). Rhodamine-labeled conA and PHA-L (Vector Laboratories; 20 μg/ml) were dissolved in blocking solution and incubated for 5 min with pre-washed live cells. For nuclear staining, Hoechst 33,342 (Thermo Fisher Scientific; 5 μM) was incubated with the live cells for 30 min. The cells were then washed, fixed in 4% PFA in PBS for 20 min, and mounted using ProLong Antifade reagent.

Quantum dot (QD) tracking of the GluN3A subunits was performed as described previously (Ferreira et al., 2017; Mikasova et al., 2017). In brief, cultured hippocampal neurons (days *in vitro*, DIV13–15) expressing the GFP-GluN3A subunit were washed in pre-warmed Neurobasal Medium (Thermo Fisher Scientific) containing 1% (w/v) bovine albumin serum (BSA; Merck) and then incubated for 10 min with rabbit anti-GFP antibody (Merck, 1:2000), followed by 10 min incubation with an anti-rabbit IgG conjugated to QD605 (Thermo Fisher Scientific; 1:10 000); both the primary and secondary antibodies were diluted in Neurobasal Medium containing 1% BSA and were incubated with the neurons at 37°C. After QD labeling, the neurons were washed extensively with pre-warmed Neurobasal Medium and then placed in extracellular recording solution containing 1 mM MgCl_2_ and 1 mM CaCl_2_; all recordings were performed at 37°C within 30 min of QD labeling. QD-labeled GluN3A subunits were detected using the InsightSSI illumination module, an oil immersion objective (60× 1.42, PlanApo N), and excitation/emission filters (excitation 571/19, emission 609/37) on a wide-field fluorescence microscope (DeltaVision OMXTM). Images (up to 1200 consecutive frames) were obtained with an acquisition time of 50 ms. As a negative control for lateral mobility, COS-7 cells transfected with GluN1–4a/GFP-GluN3A receptors were labeled with a rabbit anti-GFP primary antibody and a QD605-conjugated anti-rabbit IgG and then fixed with PFA in PBS as described above. QD movements were analyzed in ImageJ using the Mosaic Particle Tracker 2D/3D plug-in (Sbalzarini and Koumoutsakos, 2005). For each QD trajectory, the short-range diffusion coefficient, D, was obtained from the linear fit of the first four points of the computed mean-squared displacement (MSD) vs. time delay *t* as follows: *MSD*_t_ = 4 Dt + b (Kusumi et al., 1993; Triller and Choquet, 2008). Out of the total 1254 analyzed trajectories, 10 trajectories gave a negative slope in the MSD linear fit. These 10 trajectories were assigned the diffusion coefficient *D* = 0. The cumulative probability distributions were computed as the relative cumulative frequency of D from all trajectories in the indicated experiment.

### Deglycosylation and Lectin-Based Biochemical Assays

Deglycosylation experiments were performed using rat cultured hippocampal neurons solubilized for 30 min at 37°C in 1% (w/v) sodium deoxycholate in 50 mM Tris-HCl containing 1 mM EDTA and protease inhibitors (Roche; pH 7.3), followed by centrifugation at 30,000× *g* for 30 min at 4°C. The hippocampal lysates were then denatured in Denaturing Buffer (New England Biolabs) at 100°C for 10 min in accordance with the manufacturer’s protocol. Transfected HEK293 cells were directly collected in Denaturing Buffer. For peptide-*N*-glycosidase F (PNGase F) treatment, 10× G7 reaction buffer, 10% (v/v) Nonidet P-40, and 500 units of PNGase F were added. For endoglycosidase H (Endo H) treatment, 10× G5 reaction buffer and 1000 units of Endo H were added. Experiments using *O*-glycosidase and neuraminidase (both from New England Biolabs) were formed in accordance with the manufacturer’s protocol. The samples were incubated at 37°C either for 2 h or overnight, then heated for 3 min at 80°C in 2× sodium dodecyl sulfate (SDS) loading buffer.

For the lectin association experiments, 800 μg of total protein lysates from rat cultured hippocampal neurons or transfected HEK293 cells (prepared as described above) was incubated with biotinylated/agarose-linked lectin (40 μl; Vector Laboratories) with rotation at 4°C overnight. The samples containing biotinylated lectins were then incubated with 40 μl of streptavidin-coated agarose beads (Vector Laboratories) for 2 h at room temperature. The samples were washed three times with wash buffer containing 0.1% (w/v) TX-100 in Tris-buffered saline (pH 7.4), and then heated for 3 min at 80°C in 2× SDS loading buffer (25 μl). The proteins were separated by electrophoresis in a 5% or 7% SDS-polyacrylamide gel (SDS-PAGE), transferred to a polyvinylidene difluoride (PVDF) membrane, then incubated with the respective primary and secondary antibodies. The signal was detected using enhanced chemiluminescence with BioMax MR X-ray film (Eastman Kodak). The intensity of the protein bands was quantified using ImageJ software and normalized to the values obtained from the respective negative control.

### Drugs, Enzymes, Lectins and Antibodies

The following inhibitors of *N*-glycosylation were used in this study: tunicamycin (Tocris), 1-deoxynojirimycin (DNJ; Merck), kifunensine (Merck), DMM (Tocris), and swainsonine (Tocris); the final concentration of DMSO in the culture media was <0.1%. PNGase F (10,000 U/ml) was applied to live HEK293 cells in PBS containing 1 mM MgCl_2_ and 0.1 mM CaCl_2_ at 37°C as described previously (Lichnerova et al., 2015). Unconjugated conA, WGA and AAL (Vector Laboratories), which were used in QD tracking experiments, were dissolved in accordance with the manufacturer’s recommendations; the stock solutions were diluted to a final concentration of 20 μg/ml in extracellular recording solution prior to imaging. The following primary antibodies were used for biochemistry: rabbit anti-GluN3A (1:1000; Merck), anti-GFP (1:1000; Merck), and anti-GluN1 (clone 54.2; 1:1000; from Dr. Wenthold, NIDCD/NIH); the following secondary antibodies were used: horseradish peroxidase (HRP)—conjugated donkey anti-rabbit and donkey anti-mouse (1:1000; Amersham). The following primary antibodies were used for fluorescence microscopy: rabbit anti-GFP (1:1000; Merck), mouse anti-GFP (1:500, Abcam), mouse anti-protein disulfide isomerase (PDI) (1:200; Abcam), rabbit anti-Golgi matrix protein 130 (GM130) (1:500; Merck), and mouse anti-postsynaptic density protein 95 (PSD-95) (1:500; Merck); the following secondary antibodies were used: Alexa Fluor 488/647-conjugated goat anti-mouse and goat anti-rabbit (1:1000; Thermo Fisher Scientific).

### Statistical Analysis

Group differences were analyzed using the unpaired Student’s *t*-test or one-way ANOVA followed by the Student-Newman-Keuls test, where indicated in the figure legends. The distributions of diffusion coefficients were compared using the Mann-Whitney-Wilcoxon test and the Kolmogorov-Smirnov test.

## Results

### *N*-Glycosylation at Specific Sites Regulates the Surface Delivery of GluN3A-Containing NMDARs in Mammalian Cell Lines and Hippocampal Neurons

**Figure 1:**
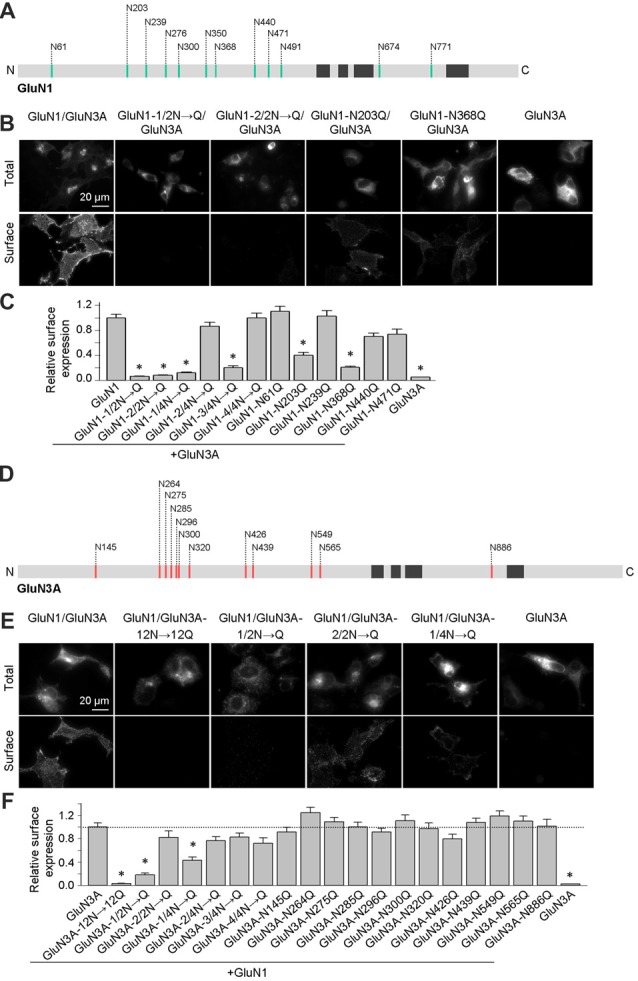
Altered surface expression of GluN1/GluN3A receptors lacking specific *N*-glycosylation sites. **(A,D)** Schematic diagram of the 12 consensus *N*-glycosylation sites (N-X-S/T) in the GluN1 **(A)** and GluN3A **(D)** subunits. N = N-terminus; C = C-terminus; black rectangles indicate membrane domains. **(B,E)** Representative images of total and surface wild-type and mutant GluN1–4a/GFP-GluN3A (hereafter referred to as GluN1/GluN3A) receptors or GluN3A subunits expressed in COS-7 cells, immunostained 24 h after transfection. **(C,F)** Summary of the relative surface expression of the indicated NMDAR subunits measured using fluorescence microscopy (*n* ≥ 54 cells per group). *p < 0.05 vs. GluN1/GluN3A (ANOVA). In this and subsequent figures, all summary data are presented as the mean ± SEM.

We previously reported that two *N*-glycosylation sites in the GluN1 subunit (specifically, N203 and N368) regulate the release of GluN1/GluN2 receptors from the ER (Figure 1A; Lichnerova et al., 2015). Here, we first examined whether specific *N*-glycosylation site(s) in GluN1 and/or GluN3A are required for the trafficking of GluN3A-containing NMDARs to the cell surface. Our electrophysiological measurements in transfected HEK293 cells showed that co-expressing the GluN1–4a splice variant with GluN3A produces larger glycine-induced currents compared to co-expressing the GluN1–1a subunit (data not shown); this finding is consistent with previous reports (Smothers and Woodward, 2009; Cummings and Popescu, 2016). Therefore, we generated mutant versions of the GluN1–4a subunit (hereafter referred to as GluN1) in which various combinations of the 12 consensus *N*-glycosylation sites were replaced with a glutamine residue (N→Q). We then expressed these subunits in COS-7 cells and measured both total and surface expression. We found that replacing the first six *N-*glycosylation sites (i.e., 1–6; GluN1–1/2N→Q) or the second six sites (i.e., 7–12; GluN1–2/2N→Q) significantly reduced the surface delivery of the resulting GluN1/GluN3A NMDARs (Figures 1B,C). Interestingly, mutating various triplet sets of *N*-glycosylation sites in GluN1–4a either had no effect on surface delivery (GluN1–2/4N→Q and GluN1–4/4N→Q) or significantly reduced the surface delivery (GluN1–1/4N→Q and GluN1–3/4N→Q) of the resulting GluN1/GluN3A receptors (Figures 1B,C). This suggests that specific *N*-glycosylation sites in the GluN1–4a subunit regulate the delivery of GluN1/GluN3A receptors to the cell surface. Next, we mutated each of the six *N*-glycosylation sites in the GluN1–1/4N→Q and GluN1–3/4N→Q subunits and expressed each construct together with the GluN3A subunit in COS-7 cells. These experiments revealed that *N-glycosylation* at both N203Q and N368Q is essential for receptor trafficking to the cell surface; mutating either of these two sites significantly reduced surface expression of GluN1/GluN3A NMDARs (Figure 1C). Thus, these two specific *N*-glycosylation sites in the GluN1 subunit regulate the surface delivery of both GluN1/GluN2 NMDARs and GluN1/GluN3A NMDARs.

**Figure 2:**
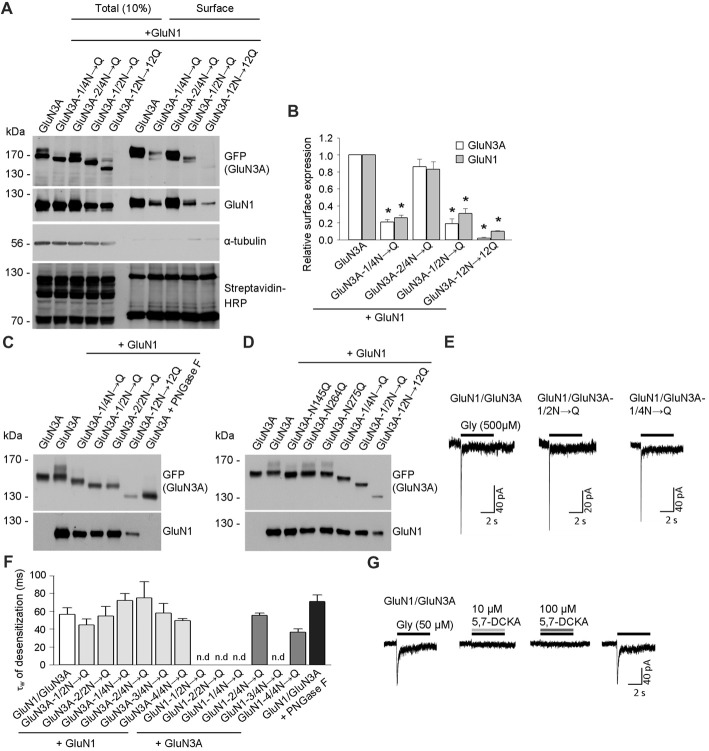
Biochemical and electrophysiological characterization of GluN1/GluN3A receptors lacking specific *N-*glycosylation sites. **(A)** Cell-surface biotinylation assay for the indicated GFP-GluN3A subunits co-expressed with GluN1–4a (GluN1) in HEK293 cells. The surface receptors were biotinylated and then pulled down using Streptavidin Agarose Resin. Total input (10% of the lysate) and surface GluN3A or GluN1 subunits were then detected using anti-GFP and anti-GluN1 antibodies, respectively; α-tubulin was used as a loading control and as a negative control for surface labeling; Streptavidin-horseradish peroxidase (HRP) blot shows control surface biotinylation levels. **(B)** Summary of the experiments shown in **(A)**; the band intensity of the surface and total*N*-methyl-D-aspartate receptor (NMDAR) pools was quantified using ImageJ software and is expressed relative to wild-type GluN1/GluN3A (*n* = 3); *p < 0.05 vs. wild-type GluN1/GluN3A (Student’s *t*-test). **(C,D)** HEK293 cells were co-transfected with either wild-type or the indicated mutant GFP-GluN3A (GluN3A) subunits together with the GluN1–4a subunit (GluN1); 36 h after transfection, the GluN3A subunit was analyzed using 7% **(C)** or 5% **(D)** Sodium dodecyl sulfate (SDS)-PAGE followed by western blot analysis. For comparison, the mobility of de-glycosylated GluN3A is shown for HEK293 cells treated overnight with peptide-N-glycosidase F (PNGase F; **C**). **(E,F)** Whole-cell patch-clamp recordings were performed in HEK293 cells transfected with the indicated GluN1/GluN3A subunits. Currents were elicited by application of 500 μM glycine for 5 s (indicated by the horizontal bar in **E**). **(F)** Summary of the τ_w_ of desensitization values for the indicated GluN1/GluN3A receptors; *n* > 5, *p* >0.05 (ANOVA); n.d. = not detectable. **(G)** Whole-cell patch-clamp recordings were performed in HEK293 cells transfected with the wild-type GluN1/GluN3A subunits. Currents were elicited by application of 50 μM glycine (indicated by the black horizontal bar), the co-application of a competitive antagonist of the NMDARs, 10 μM or 100 μM 5,7-dichlorokynurenic acid (5,7-DCKA; indicated by the gray horizontal bars) resulted in complete inhibition of the GluN1/GluN3A receptor-mediated currents.

Similar to the GluN1 subunit, the GluN3A subunit also contains 12 consensus *N*-glycosylation sites (Figure 1D). We therefore systematically mutated the 12 sites in GluN3A using a strategy similar to the GluN1–4a subunit and measured total and surface expression of the resulting GluN1/GluN3A receptors expressed in COS-7 cells (Figures 1E,F). We found that replacing all 12 *N*-glycosylation sites in the GluN3A subunit (GluN3A-12N→12Q) virtually eliminated the surface expression of GluN1/GluN3A receptors. Furthermore, mutating the first six sites (GluN3A-1/2N→Q), but not the second six sites (GluN3A-2/2N→Q), also reduced the surface expression of GluN1/GluN3A receptors. Interestingly, similar to our screen of the GluN1–4a subunit, mutating various triplet *N*-glycosylation sites in the GluN3A subunit either had no effect (GluN3A-2/4N→Q, GluN3A-3/4N→Q, and GluN3A-4/4N→Q) or significantly reduced the surface delivery (GluN3A-1/4N→Q) of GluN1/GluN3A receptors (Figures 1E,F). Lastly, we mutated each of the 12 *N*-glycosylation sites in the GluN3A subunit and co-expressed each mutant together with GluN1–4a in COS-7 cells. These experiments revealed that no single *N-glycosylation* site in GluN3A is essential for the surface delivery of GluN1/GluN3A receptors (Figures 1E,F). Together, our experiments showed that simultaneous glycosylation of all three key asparagine residues—N145, N264, and N275—in the GluN3A subunit is required for surface delivery of GluN1/GluN3A receptors in cultured mammalian cells; this conclusion is supported by a biotinylation assay in transfected HEK293 cells (Figures 2A,B).

To confirm that these three asparagine residues in the GluN3A subunit are indeed occupied by *N*-glycans, we co-expressed the mutant GluN3A subunits together with the GluN1 subunit in HEK293 cells and analyzed the proteins using a gel-shift assay (Figures 2C,D). Interestingly, the gel mobility of the GluN3A-12N→12Q subunit (with all 12 *N*-glycosylation sites mutated) was similar to the wild-type GluN3A subunit treated with PNGase F, which removes all types of *N*-glycans from glycoproteins, indicating that many of the 12 conventional *N*-glycosylation sites in GluN3A are indeed glycosylated. We also examined other mutant GluN3A subunits and found that gel mobility was shifted (relative to wild-type) for GluN3A subunits lacking either six (GluN3A-1/2N→Q; GluN3A-2/2N→Q) or three (GluN3A-1/4N→Q) *N*-glycosylation sites (Figure 2C). Lastly, we examined the effect of mutating each of the three sites identified as essential for surface delivery and found that all three single mutant subunits (i.e., N145Q, N264Q and N275Q) had altered gel mobility relative to the wild-type GluN3A subunit; in addition, the GluN3A-12N→12Q, GluN3A-1/2N→Q and GluN3A-1/4N→Q mutants had altered mobility as well (Figure 2D). Taken together, these results confirm that three specific asparagine residues (N145, N264, and N275) in the GluN3A subunit are indeed *N*-glycosylated when expressed in HEK293 cells.

To examine the functional relevance of these *N*-glycosylation sites in GluN subunits, we examined whether GluN1–4a and/or GluN3A subunits lacking specific *N-*glycosylation sites can form functional channels at the cell surface. We therefore co-transfected HEK293 cells with wild-type or mutant GluN1–4a subunits together with wild-type or mutant GluN3A subunits and recorded whole-cell currents induced by rapid application of 500 μM glycine (Figures 2E,F). We observed robust glycine-induced currents in the majority of the GluN1–4a/GluN3A subunit combinations examined, whereas no detectable currents were measured when cells expressed GluN1–1/2N→Q/GluN3A, GluN1–2/2N→Q/GluN3A, GluN1–1/4N→Q/GluN3A, or GluN1–3/4N→Q/GluN3A receptors (Figures 2E,F). These findings are consistent with our microscopy and biochemistry data, confirming that these specific NMDARs are not delivered to the cell surface. Our analysis of the currents mediated by wild-type GluN1/GluN3A receptors showed a weighted time constant for desensitization (τ_w_ of desensitization) of approximately 55 ms, consistent with previous studies regarding this receptor subtype (Cummings and Popescu, 2016); moreover, similar τ_w_ values were measured in the subunit combinations that yielded detectable currents (Figures 2E,F). Indeed, the co-application of a competitive antagonist 5,7-dichlorokynurenic acid (5,7-DCKA; 10 μM or 100 μM) resulted in complete inhibition of the GluN1/GluN3A receptor-mediated currents (Figure 2G). Thus, our electrophysiological experiments indicate that the removal of most of the *N*-glycosylation sites, including the GluN3A-1/4N→Q mutant, did not affect the ability to form functional GluN1/GluN3A receptors at the cell surface. This conclusion is supported by our analysis of HEK293 cells incubated with a high concentration of PNGase F, which targets *N*-glycans at the cell surface (but does not affect delivery of the subunit to the cell surface); treating cells that express wild-type GluN1/GluN3A receptors with PNGase F did not significantly affect the τ_w_ of desensitization (Figure 2F).

**Figure 3:**
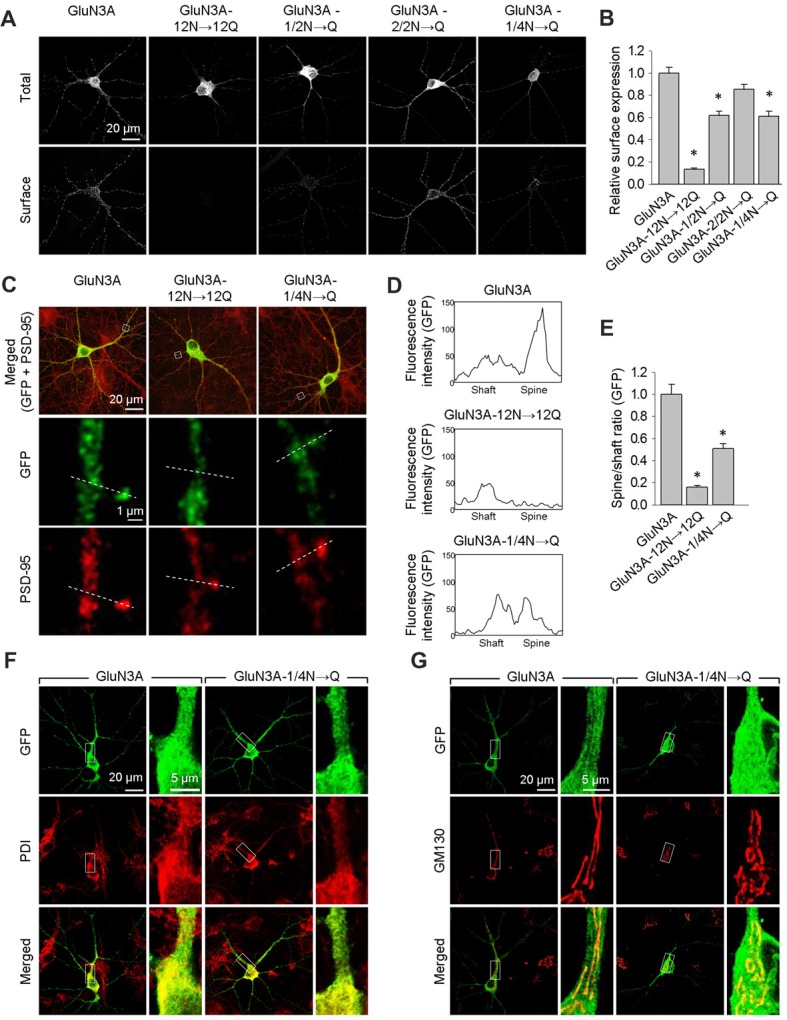
The GluN3A-1/4N→Q subunit has reduced surface expression in hippocampal neurons. **(A)** At days *in vitro* (DIV)10, cultured hippocampal neurons were transfected with the indicated GFP-GluN3A (GluN3A) subunits; at DIV14, the cells were immunostained for surface and total GFP under non-permeabilizing and permeabilizing conditions, respectively. Representative images of total and surface immunoreactivity are shown. **(B)** Summary of the relative surface expression of the indicated GluN3A subunits obtained from 10-μm long segments of secondary and tertiary dendrites (*n* ≥ 36 segments from ≥12 neurons for each GluN3A subunit); *p < 0.05 vs. wild-type GluN3A (ANOVA). **(C–E)** Cultured hippocampal neurons were transfected with the indicated GluN3A subunit at DIV10; at DIV14, the cells were immunostained under permeabilizing conditions using anti-GFP and anti-postsynaptic density protein 95 (PSD-95) antibodies; PSD-95 staining was used to visualize the dendritic spines. ** (C)** Representative images of neurons showing co-localization between GFP and PSD-95 immunoreactivity in cells transfected with indicated the GluN3A subunits. The dashed lines were used to generate line plots for a spine and an adjacent dendritic shaft for GFP (middle row) and PSD-95 fluorescence (bottom row). **(D)** Representative graphs of the intensity of the GFP signal in a dendritic shaft and spine of neurons transfected with the indicated GluN3A subunits. **(E)** Summary of the ratio of spine/shaft fluorescence intensity calculated from the peak fluorescence intensity measured in spine and shaft pairs (*n* = 30 spines from ≥5 neurons for each GluN3A subunit); *p < 0.05 vs. GluN3A (ANOVA). There was no significant difference between the wild-type and mutant GluN3A with respect to GFP intensity in dendritic shafts (data not shown). **(F,G)** Hippocampal neurons were transfected with the indicated GluN3A subunits on DIV10; at DIV14, the cells were immunostained for the endoplasmic reticulum (ER) marker protein disulfide isomerase (PDI; **F**) or the GA marker Golgi matrix protein (GM130; **G**).

Next, we expressed wild-type or mutant forms of GluN3A in cultured hippocampal neurons and measured their surface expression. Consistent with our results obtained with COS-7 cells, neurons that express the GluN3A-12N→12Q, GluN3A-1/2N→Q, or GluN3A-1/4N→Q subunits—but not cell that express GluN3A-2/2N→Q subunits—have reduced surface expression relative to wild-type GluN3A subunits (Figures 3A,B). We then performed co-immunofluorescence experiments in hippocampal neurons expressing either wild-type or mutant GluN3A subunits, using antibodies against PSD-95 (an excitatory synapse marker), PDI (an ER marker), or GM130 (a Golgi apparatus (GA) marker). To measure the delivery of GluN3A subunits to the dendritic surface, we generated line plots of the fluorescence intensity of labeled GluN3A, GluN3A-12N→12Q, and GluN3A-1/4N→Q subunits through the spine heads and the surrounding dendritic shafts (Figures 3C–E). We then calculated the ratio of the peak intensity in the spine to the peak intensity in the shaft, as described previously (Gerges et al., 2004). Interestingly, we found that the spine/shaft ratio was significantly lower in neurons expressing the GluN3A-1/4N→Q subunit compared to neurons expressing wild-type GluN3A. The ratio was even lower in neurons expressing the GluN3A-12N→12Q subunit, supporting our conclusion that this subunit does not reach the cell surface.

Interestingly, we found that both the wild-type and GluN3A-1/4N→Q subunits co-localize with the ER marker PDI when overexpressed in neurons (Figure 3F). This finding may be explained by a limiting number of available endogenous GluN1 subunits in these neurons (Prybylowski et al., 2002). Confocal microscopy also revealed that neither wild-type GluN3A nor the GluN3A-1/4N→Q subunit accumulates in the GA (Figure 3G). Given that the number of GluN3A-1/4N→Q subunits was significantly reduced at both the cell surface and dendritic spines and given that this subunit does not accumulate in the GA, we propose that the GluN3A-1/4N→Q subunit is retained in the ER to a greater extent than the wild-type GluN3A subunit. Taken together, these data indicate that the N*-*terminal *N*-glycosylation sites in the GluN3A subunit are essential for the forward trafficking of GluN3A-containing NMDARs in hippocampal neurons.

### Biochemical Characterization of the *N-*Glycans in GluN3A-Containing NMDARs

**Figure 4:**
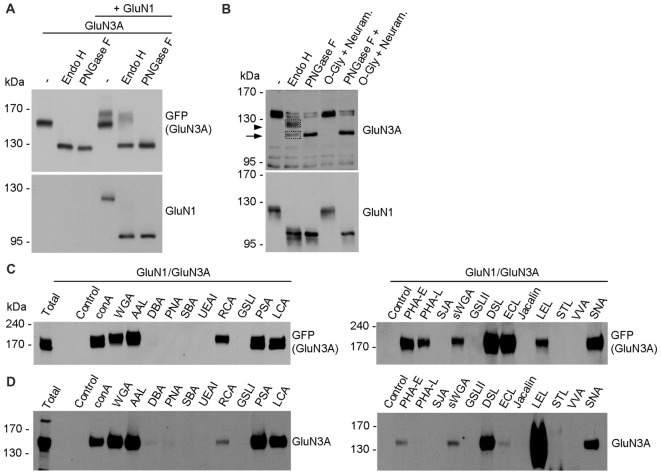
Biochemical analysis of GluN3A-containing NMDARs. **(A,B)** HEK293 cells expressing GluN1/GluN3A receptors **(A)** and cultured hippocampal neurons at DIV14 **(B)** were treated overnight with PNGase F or endoglycosidase H (Endo H), then subjected to western blot analysis using anti-GFP (for GFP-GluN3A), anti-GluN3A, or anti-GluN1 antibodies. Representative immunoblots are shown. The arrowhead in B indicates the Endo H-resistant “mature” GluN3A subunit, and the arrow indicates the Endo H-sensitive “immature” GluN3A subunit. **(C,D)** Lysate were prepared from transfected HEK293 cells **(C)** and cultured hippocampal neurons at DIV14 **(D)** and incubated with the indicated biotinylated lectins overnight, then precipitated with Streptavidin Agarose Resin for 2 h at 4°C. The proteins were then separated by SDS-PAGE, transferred to a polyvinylidene difluoride (PVDF) membrane, and probed using anti-GFP and anti-GluN3A. Representative immunoblots from three independent experiments are shown.

In general, the *N*-glycans in glycoproteins exist as high-mannose forms, which arise in the ER, or as hybrid and/or complex forms, which arise in the GA (Vagin et al., 2009; Moremen et al., 2012). To determine which forms of *N*-glycans are present on both endogenous and recombinant GluN3A-containing NMDARs, we treated cultured hippocampal neurons (DIV14) and HEK293 cell expressing GluN1/GluN3A receptors overnight with either PNGase F (which removes all forms of *N*-glycans) or Endo H (which removes only the high-mannose and hybrid forms of *N*-glycans); we then measured the mobility of GluN1 and GluN3 subunits using SDS-PAGE (Figures 4A,B). These experiments revealed that recombinant GluN1 and GluN3A subunits co-expressed in HEK293 cells have similar electrophoretic mobility regardless of whether the cells were treated with PNGase F or Endo H (Figure 4A). Similarly, the electrophoretic mobility of Endo H-treated endogenous GluN1 subunits was similar to the electrophoretic mobility of PNGase F-treated endogenous GluN1 subunits, consistent with previous reports (Huh and Wenthold, 1999). In contrast, Endo H treatment had a different effect on the electrophoretic mobility of endogenous GluN3A subunits compared to PNGase F treatment, indicating that the *N*-glycans present on endogenous GluN3A subunits are complex *N*-glycans. Interestingly, a small fraction of GluN3A subunits was completely insensitive to both PNGase F and Endo H treatment, showing no change in electrophoretic mobility even after the cells were treated for >24 h (data not shown); this fraction of GluN3A subunits was also unaffected by both neuraminidase and *O*-glycosidase treatment (Figure 4B), suggesting that this resistant fraction of GluN3A subunits is neither *N*-glycosylated nor *O*-glycosylated.

Next, we asked which *N*-glycan structures are incorporated into recombinant and endogenous GluN3A subunits using a comprehensive panel of conjugated lectins. Detailed information regarding the glycan specificities of the lectins tested is provided in Table 1. After precipitating specific lectin-bound proteins from lysates prepared from HEK293 cells and cultured hippocampal neurons, we measured the relative levels of the GluN3A subunit using western blot analysis (Figures 4C,D). Interestingly, the GluN3A subunits in both cell types were strongly pulled down with lectins that recognize high-mannose, hybrid, and complex *N-*glycans, including conA, WGA, AAL, PSA, LCA, DSL and SNA. The differences observed in lectin sensitivity (e.g., RCA, PHA-E, ECL and LEL) between HEK293 cells and hippocampal neurons likely reflect differences in the *N-*glycosylation machinery between these two cell types. Taken together, these data indicate that both recombinant and endogenous GluN3A subunits contain a broad diversity of *N*-glycan structures, including hybrid and/or complex forms.

**Table 1:** Glycan specificities of the lectins used for the immunoprecipitation experiments.

Shortcut	Lectin	Preferred glycan specifity^1^
ConA	*Concanavalin A*	αMan
WGA	*Wheat germ agglutinin*	GlcNAc
AAL	*Aleuria aurantia*	Fucα_6_GlcNAc
DBA	*Dolichos biflorus*	αGalNAc
PNA	*Peanut*	Galβ_3_GalNAc
SBA	*Soybean*	α/βGalNAc
UEIA	*Ulex europaues*	αFuc
RCA	*Ricinus communis*	Gal
GSL I	*Griffonia simplicifolia I*	αGal/αGalNAc
PSA	*Pisum sativum*	αMan
LCA	*Lens culinaris*	αMan
PHA-E	*Phaseolus vulgaris Erythroagglutinin*	Galβ_4_GlcNAcβ_2_Manα_6_(GlcNAcβ4) (GlcNAcβ_4_Manα_3_), Manβ_4_
PHA-L	*Phaseolus vulgaris Leucoagglutinin*	Galβ_4_GlcNAcβ_6_(GlcNAcβ_2_Manα_3_)Manα_3_
SJA	*Sophora japonica*	βGalNAc
sWGA	succinylated *wheat germ agglutinin*	GlcNAc
GSL II	*Griffonia simplicifolia II*	α/βGlcNAc
DSL	*Datura stramonium*	(GlcNAc)_2-4_
ECL	*Erythrina crystagalli*	Galβ_4_GlcNAc
Jacalin	*Jacalin*	Galβ3GalNAc
LEL	*Lycopersicon esculentum*	(GlcNAc)_2-4_
STL	*Solanum tuberosum*	(GlcNAc)_2-4_
VVA	*Vicia villosa*	α/βGalNAc
SNA	*Sambucus nigra*	Neu_5_Acα_6_Gal/GalNAc

*Adapted from the Vector Laboratories manual and from Varki et al. (2009). See Figure 4 for further details. ^1^Man, mannose; GlcNAc, *N*-acetylglucosamine; Gal, galactose; GalNAc, *N*-acetylgalactosamine; Fuc, fucose; Neu5Ac, *N*-acetylneuraminic acid*.

### *N-*Glycan Remodeling Is Not Required for Surface Delivery of GluN3A-Containing NMDARs

**Figure 5:**
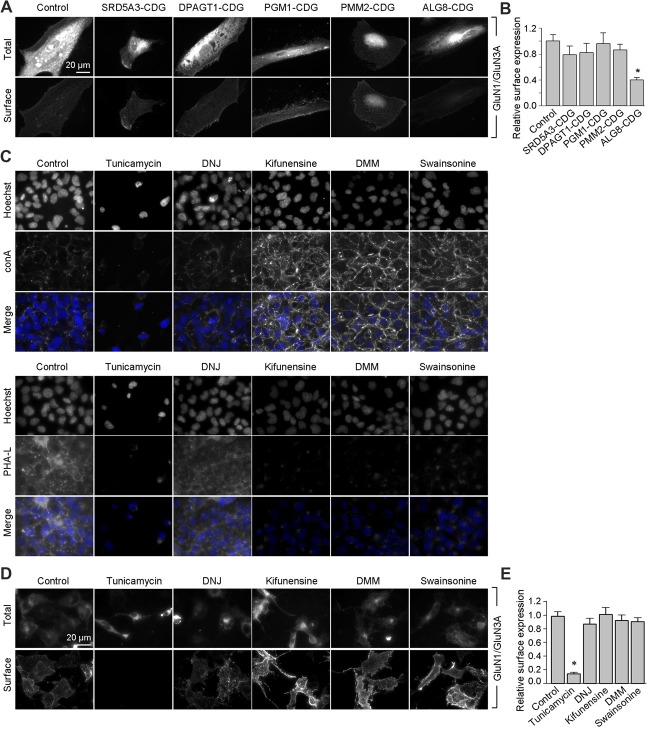
Remodeling of *N*-glycans is not essential for surface delivery of recombinant GluN1/GluN3A receptors. **(A)** The indicated human fibroblasts were transfected with GluN1 and GluN3A. Detailed information regarding the fibroblasts used here is provided in Table 2. Representative images of total and surface pools of labeled subunits 24 h after transfection are shown. **(B)** Summary of the normalized surface expression of GluN1/GluN3A receptors measured using fluorescence microscopy; *n* ≥ 20 cells from two independent experiments. *p < 0.05 vs. the GluN1/GluN3A signal in the control group (ANOVA). **(C)** COS-7 cells were treated for 2 days with 1 μg/ml tunicamycin, 200 μg/ml 1-deoxynojirimycin (DNJ), 5 μg/ml kifunensine, 200 μg/ml 1-deoxymannojirimycin (DMM), or 100 μg/ml swainsonine. The cells were then labeled with fluorescently tagged conA or PHA-L and Hoechst 33342 (Hoechst); representative images are shown. **(D)** COS-7 cells were transfected with GluN1 and GluN3A and then treated with the *N*-glycosylation inhibitors as described above. Representative images of total and surface pools of labeled GluN1/GluN3A receptors are shown. **(E)** Summary of the normalized surface expression of GluN1/GluN3A receptors measured using fluorescence microscopy; *n* ≥ 30 cells. *p < 0.05 vs. the control group (ANOVA).

Next, we asked whether remodeling of *N*-glycans affects the trafficking of GluN3A-containing NMDARs to the cell surface. First, we expressed recombinant GluN1 subunits in human fibroblast cell lines derived from patients with various congenital disorders of glycosylation (CDG; see Table 2), as well as control cells, and measured the surface expression of GluN1/GluN3A receptors (Figure 5A). These experiments revealed that control fibroblasts and four of the five CDG fibroblast lines have robust surface delivery of GluN1/GluN3A receptors (Figures 5A,B; Table 2). In contrast, the ALG8-CDG line, which has reduced formation of *N-*glycans in the ER, had significantly reduced surface delivery of GluN1/GluN3A receptors. Thus, reduced *N-*glycosylation within the ER—but not altered *N-*glycan remodeling—reduces the delivery of GluN1/GluN3A receptors to the cell surface.

**Table 2:** Details regarding the fibroblasts obtained from patients with the indicated CDG syndromes.

Type	Gene mutation(s)	Protein mutations	Reference
SRD5A3-CDG	homozygous c.[436G >A]	p.E146K	Honzik et al. (2013)
DPAGT1-CDG	c.[85A >T];[652C >T]	p.[I29F];[R218W]	Honzik et al. (2013)
PGM1-CDG	c.[1010C >T];[1508G >A]	p.[T337M];[R503Q]	Ondruskova et al. (2014)
PMM2-CDG	c.[422G >A];[691G >A]	p.[R141H];[V231M]	-
ALG8-CDG	c.[139A >C];[1090C >T]	p.[T47P];[R364X]	Vesela et al. (2009)

*CDG, congenital disorders of glycosylation; SRD5A3, steroid 5-alpha-reductase 3; DPAGT1, dolichyl-phosphate alpha-N-acetylglucosaminyltransferase; PGM1, phosphoglucomutase 1; PMM2, phosphomannomutase 2; ALG8, alpha-1,3-glucosyltransferase*.

Next, we asked whether inhibiting the *N*-glycosylation machinery affects the surface delivery of GluN3A-containing NMDARs. We first incubated COS-7 cells for 2 days in the presence or absence of various inhibitors, including 1 μg/ml tunicamycin (an inhibitor of dolichyl-phosphate *N-acetylglucosamine phosphotransferase* 1; Yavin et al., 1984), 200 μg/ml DNJ (an inhibitor of α-glucosidase I-II; Gross et al., 1986), 5 μg/ml kifunensine (an inhibitor of the ER α-mannosidase I; Herreman et al., 2003), 200 μg/ml DMM (an inhibitor of the ER α-mannosidase I-II; Tokhtaeva et al., 2009), or 100 μg/ml swainsonine (an inhibitor of the GA α-mannosidase II; Liu et al., 2000). With the exception of DNJ, the other four inhibitors drastically reduced surface labeling with fluorescently tagged PHA-L (which recognizes complex types of *N-*glycans; Figure 5C). In contrast, with the exception of tunicamycin, the other four inhibitors increased surface labeling with fluorescently tagged conA (which recognizes the high-mannose forms of *N-*glycans; Figure 5C). These results indicate that treating COS-7 cells with tunicamycin, kifunensine, DMM, and swainsonine inhibits the *N-*glycosylation machinery.

**Figure 6:**
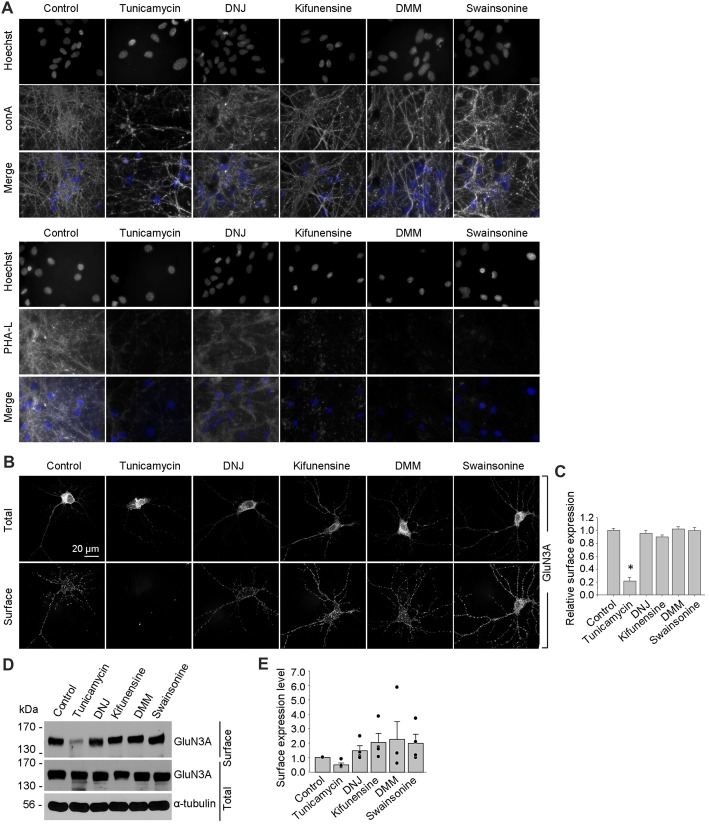
Tunicamycin, but not inhibitors of later step in the *N*-glycosylation pathway, reduces the surface delivery of GluN3A subunits in hippocampal neurons. **(A)** Hippocampal neurons were treated for 2 days with 0.5 μg/ml tunicamycin, 200 μg/ml DNJ, 5 μg/ml kifunensine, 200 μg/ml DMM, or 100 μg/ml swainsonine. The neurons were then labeled with fluorescently tagged conA or PHA-L and Hoechst 33342 (Hoechst); representative examples are shown. **(B)** Hippocampal neurons were transfected with GFP-GluN3A (GluN3A) at DIV8 and treated for 2 days with the indicated inhibitors as described above. The neurons were then immunostained for surface and total GFP under non-permeabilizing and permeabilizing conditions, respectively; representative images of total and surface immunoreactivity are shown. **(C)** Summary of the relative surface expression of GluN3A subunits in neurons treated with the indicated inhibitors; data were obtained from 10-μm long segments of secondary and tertiary dendrites (*n* ≥ 30 segments from ≥6 neurons) and are expressed relative to control-treated neurons; *p < 0.05 vs. control (ANOVA). **(D)** A surface biotinylation assay was performed at DIV15 on dense cultures of hippocampal neurons treated for 2 days with the indicated inhibitors applied at the concentrations described above. **(E)** Graph of the relative surface expression measured using the surface biotinylation assay shown in **(D)**; the intensity of the surface and total GluN3A bands were quantified using ImageJ; the black dots indicate the values obtained in the individual experiments; *n* = 4. *p < 0.05 vs. control (Student’s *t*-test).

Using these inhibitors in transfected COS-7 cells, we found reduced surface delivery of GluN1/GluN3A receptors in cells treated with tunicamycin; in contrast, DNJ, kifunensine, DMM, and swainsonine had no effect on the surface delivery of GluN1/GluN3A receptors (Figures 5D,E). These data support our hypothesis that the initial attachment of *N-*glycans in the ER—but not the subsequent remodeling of *N-*glycan composition—is required for surface delivery of GluN1/GluN3A receptors. Next, we treated cultured hippocampal neurons for 2 days with 0.5 μg/ml tunicamycin (this slightly reduced concentration was used because 1 μg/ml was toxic to these neurons; data not shown) or the same concentrations of DNJ, kifunensine, DMM, and swainsonine listed above. These experiments revealed a pattern of labeling with fluorescently tagged conA and PHA-L that was similar to our results obtained with COS-7 cells (Figure 6A), confirming that four of the five inhibitors effectively impaired the *N-*glycosylation machinery. Finally, we found that tunicamycin—but not DNJ, kifunensine, DMM, or swainsonine—drastically reduced the surface delivery of the recombinant GFP-GluN3A subunit in hippocampal neurons (Figures 6B,C). Similar results were obtained with respect to the surface delivery of endogenous GluN3A subunits measured in hippocampal neurons treated with these five inhibitors (Figures 6D,E). Taken together, these data indicate that remodeling of *N-*glycans does not play an essential role in the surface delivery of GluN3A-containing NMDARs.

### The Lateral Mobility of GluN3A Subunits at the Cell Surface In Hippocampal Neurons Is Bi-directionally Regulated By *N*-Glycosylation

**Figure 7:**
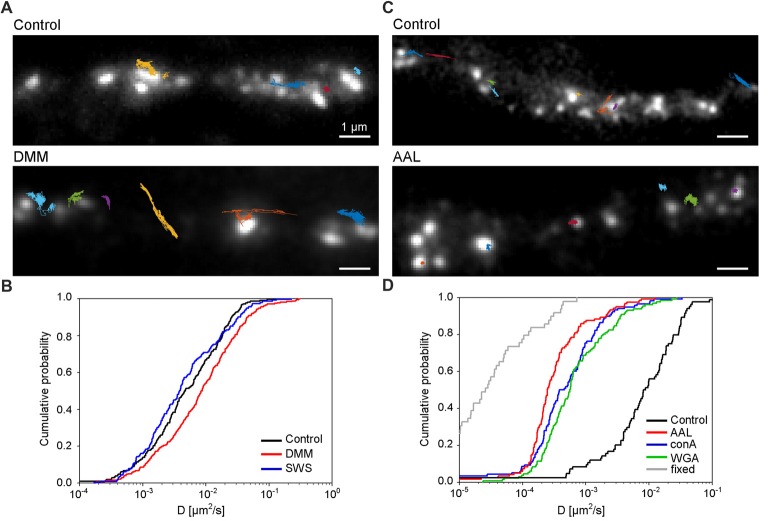
*N*-glycosylation regulates the mobility of GluN3A-containing NMDARs at the cell surface of hippocampal neurons. **(A,C)** Representative trajectories of quantum dot (QD)-labeled GFP-GluN3A (GluN3A) subunits in hippocampal neurons treated with 200 μg/ml DMM **(A)** or with the lectin AAL (20 μg/ml) **(C)**. **(B,D)** Comparison of the cumulative distribution of the diffusion coefficients measured for QD-labeled GluN3A subunits in hippocampal neurons treated for two days with 200 μg/ml DMM, 100 μg/ml swainsonine (*n* = 208–314 trajectories per condition; **B**), or incubated after QD labeling with 20 μg/ml of AAL, conA, or WGA (*n* = 84–131 trajectories per condition; **D**). The diffusion coefficients in fixed cells (shown in **D**) were obtained from an independent experiment (*n* = 49 trajectories). Pairwise comparisons of the distributions show the following statistically significant differences: DMM vs. control (*p* < 0.003), AAL, conA, WGA vs. control (*p* < 10^−23^), AAL, conA, WGA vs. fixed (*p* < 10^-13^). The largest *p-value* obtained in Kolmogorov-Smirnov test and Mann-Whitney-Wilcoxon test is stated.

Lastly, we investigated the role of *N*-glycosylation in regulating the lateral mobility of GluN3A-containing NMDARs at the surface of hippocampal neurons. First, we treated hippocampal neurons with the *N-*glycosylation inhibitors DMM and swainsonine; we then performed QD tracking of labeled GFP-GluN3A subunits (Figure 7A). The cumulative distribution of the diffusion coefficients extracted from the individual trajectories is shown in Figure 7B. Our analysis revealed that DMM—but not swainsonine—increased the diffusion coefficient of QD-labeled GluN3A subunits. This finding indicates that the presence of hybrid and/or complex *N-*glycans at the neuron’s surface regulates the mobility of GluN3A-containing NMDARs (Figure 7B). We also asked whether the mobility of QD-labeled GluN3A subunits at the surface of hippocampal neurons is affected by the presence of the three lectins found to be associated with endogenous GluN3A subunits, namely conA, WGA and AAL (see Figure 4). Interestingly, all three lectins strongly reduced the mobility of QD-labeled GluN3A subunits in hippocampal neurons (Figures 7C,D). Given that a wide variety of lectins are expressed in the mammalian CNS (see “Discussion” section), these data indicate that *N*-glycosylation plays a role in regulating the mobility of GluN3A-containing NMDARs at the surface of mammalian neurons.

## Discussion

Here, we present compelling evidence that *N*-glycosylation of the GluN3A NMDAR subunit is required for the receptor’s delivery to the cell surface, based on results obtained using both heterologous expression systems and cultured neurons. The precise mechanism by which this process is mediated is currently unknown. One possibility is that the presence of specific *N*-glycans confers a critical structure to GluN3A-containing NMDARs, thereby permitting their release from the ER (Vagin et al., 2009). Another possibility is that the GluN subunits interact with specific glycan-binding proteins such as galactose-binding lectins (Copits et al., 2014), which then facilitates the trafficking of GluN3A-containing NMDARs to the cell surface. Interestingly, our results indicate that trafficking of GluN1/GluN3A receptors is more dependent upon *N-*glycosylation of the GluN1 subunit than upon *N*-glycosylation of the GluN3A subunit. This conclusion is supported by our recent findings with respect to GluN1/GluN2 receptors (Lichnerova et al., 2015).

Our biochemical analyses revealed that the GluN3A subunit is extensively *N*-glycosylated, similar to both the GluN1 and GluN2 subunits (Clark et al., 1998; Huh and Wenthold, 1999; Kenny et al., 2009; Qiu et al., 2009; Kaniakova et al., 2012b; Lichnerova et al., 2015; Hanus et al., 2016). We found that the GluN3A subunit in rat hippocampal neurons contains a rich diversity of *N*-glycans, including hybrid and/or complex forms, which suggests that its *N*-glycan composition undergoes remodeling in the GA. In principle, the *N*-glycan composition of GluN3A-containing NMDARs could be modified in the somatic GA, dendritic Golgi outposts, and/or Golgi satellite structures (Mikhaylova et al., 2016). Indeed, *N*-glycosylation machinery is present in the dendrites of hippocampal neurons (Torre and Steward, 1996; Mikhaylova et al., 2016); thus, it is reasonable to speculate that all cellular Golgi systems play a role in the *N*-glycan processing of GluN3A-containing NMDARs. Other ionotropic glutamatergic receptors, including 2-amino-3-(5-methyl-3-oxo-1,2-oxazol-4-yl)propanoic acid (AMPA) receptors and kainate receptors, also contain a variety of *N*-glycan forms (Standley et al., 1998; Greger et al., 2003; Mah et al., 2005; Coleman et al., 2006; Hanus et al., 2016); thus, it is likely that the *N*-glycan composition of all ionotropic glutamate receptors regulates their trafficking and function *in vivo*.

Our analysis revealed differences in the sensitivity of GluN3A subunits expressed in hippocampal neurons vs. HEK293 cells to Endo H and several lectins. A likely explanation for this may be explained by differences in the *N*-glycosylation machinery between these two cell types, similarly as was shown previously for many glycoproteins in various mammalian cell types (Croset et al., 2012; Medzihradszky et al., 2015). Indeed, these findings suggest that caution should be used when selecting an experimental model for functional studies using NMDARs. On the other hand, we observed a similar pattern between hippocampal neurons and cell lines with respect to the surface expression of mutant GluN3A subunits that lack specific *N*-glycosylation sites. Similarly, we found that only tunicamycin—which inhibits the initial step in the *N*-glycosylation process—reduced the surface expression of GluN3A-containing NMDARs; this effect was similar between hippocampal neurons and cell lines. We also studied human fibroblasts obtained from patients with deficits in *N-glycosylation* machinery and found reduced surface delivery of GluN1/GluN3A receptors only in cells in which the initial step in the *N-*glycosylation pathway is impaired (i.e., ALG8-CDG). Taken together, our data suggest that the trafficking machinery in heterologous expression systems and cultured hippocampal neurons uses a common quality-control mechanism to regulate the trafficking of GluN3A-containing NMDARs.

Our finding that *N-*glycan remodeling is not required for the surface delivery of GluN3A-containing NMDARs is consistent with a recent study by Hanus et al., who reached a similar conclusion with respect to various neuronal membrane proteins (Hanus et al., 2016). In addition, we found that the mobility of QD-labeled GluN3A subunits at the surface of hippocampal neurons increases when hybrid and complex forms of *N-*glycans are removed. These findings are consistent with the report that the mobility of AMPARs can be regulated by enzymatic removal of the extracellular matrix (Frischknecht et al., 2009). In contrast, the diffusion coefficient of GluN3A subunits was reduced in the presence of lectins compared to control cells, corresponding to an approximately 4-fold increase in the time required to diffuse across a given short distance. Indeed, one possible interpretation of our data is that the lectins used in our study may indirectly affect the mobility of GluN3A-containing NMDARs by interacting with other glycoproteins at the neuronal cell surface. Because a wide variety of lectins are expressed in the mammalian CNS, including sialic acid-binding Ig-like lectins (Macauley et al., 2014) and galactose-binding lectins (Cummings et al., 2017), these lectins could affect the mobility of NMDARs *in vivo*. Other groups reported similar findings; for example, the binding of conA restricts the mobility of surface receptors for this lectin in mouse fibroblasts (Zagyansky and Edidin, 1976), and the protein galectin-3 alters the lateral mobility and clustering of β1-integrin receptors in HeLa cells (Yang et al., 2017).

The GluN3A subunit acts as a molecular brake, limiting the maturation of excitatory synapses; this subunit has also been implicated in a number of neuropsychiatric disorders (Fiuza et al., 2013; Kehoe et al., 2013, 2014). Thus, *N*-glycosylation may play as-yet unrecognized roles in the mammalian brain during health and/or disease. For example, *N*-glycosylation of the GluN3A subunit may be essential for its synaptic/perisynaptic localization and/or degradation (Pachernegg et al., 2012; Chowdhury et al., 2013). With respect to the clinical relevance of our findings, it is interesting to note that the glycosylation pattern on AMPARs is altered in the cerebral cortex of schizophrenia patients (Tucholski et al., 2013); therefore, it is reasonable to speculate that the *N*-glycosylation pattern of the GluN3A subunit may be altered in certain neurological and/or psychiatric conditions. Here we used lines with PMM2-CDG and PGM1-CDG which have defects in glycan precursor biosynthesis in cytosol, SRD5A3-CDG and DPAGT1-CDG with defect in the first steps of lipid-linked oligosaccharide synthesis (dolichol biosynthesis and cycle) on cytosolic side of ER and ALG8-CDG with defect in extension of oligosaccharide chain inside ER (references shown in Table 2). All of these defects are accompanied with neurological impairments (Freeze et al., 2015). Concerning the ALG8 deficiency (OMIM #608104), it is known that most of ALG8-deficient patients died within the first months of life. These cases were accompanied by multiple symptoms such as strong dysmorphic features, hypotonia, gastrointestinal disorders, hepatomegaly, coagulopathy, edema, cardiorespiratory problems, protein-losing enteropathy and ascites (Haeuptle and Hennet, 2009). Neurological dysfunctions included psychomotor disability, seizures, ataxia and structural abnormalities (dilatation of ventricles, corpus callosum hypoplasia, leukoencephalopathy/leukodystrophy and cortical/cerebral atrophy (Höck et al., 2015). We used fibroblast line derived from patient with mutations c.139A>C (p.T47P) and c.1090C>T (p.R364X) in ALG8. This patient was born at the 29th week of gestation complicated by oligohydramnios. Although the early postnatal adaptation was uneventful, generalized oedema, multifocal myoclonic seizures, and bleeding due to combined coagulopathy were present from the first day. Diarrhoea progressing to protein-losing enteropathy with ascites and pericardial effusion developed in the third week of life. Pharmacoresistant seizures and cortical, cerebellar and optic nerve atrophy indicated neurological involvement. The patient died at the age of 2 months owing to the progressive general oedema, bleeding and cardio-respiratory insufficiency (Vesela et al., 2009). Indeed, the altered function of the glycosylation machinery observed in patients with a CDG syndrome could result in altered surface and/or synaptic delivery of NMDARs, as many of these patients have impaired brain function (Freeze, 2006; Freeze et al., 2012; Moremen et al., 2012). Unfortunately, the availability of suitable human brain samples from patients with these relatively rare diseases is limited; however, the availability of new techniques for converting human fibroblasts into neuronal cells (Vierbuchen et al., 2010) may provide new opportunities for studying the consequences of impaired glycosylation machinery on the function of NMDARs, possibly leading to the identification of new therapeutic targets for these patients.

## Author Contributions

MH, KS and YHS designed the study. KS, SL, KL, MK and HH performed the experiments. KS, KL, MK, SL and MZ analyzed the data. YHS and MH wrote the article.

## Conflict of Interest Statement

The authors declare that the research was conducted in the absence of any commercial or financial relationships that could be construed as a potential conflict of interest.

## Abbreviations

AMPA: 2-amino-3-(5-methyl-3-oxo-1,2-oxazol-4-yl) propanoic acid
BSA: bovine albumin serum
CDG: congenital disorders of glycosylation
COS-7: African Green Monkey kidney fibroblast cell line
DIV: day *in vitro*
DNJ: 1-deoxynojirimycin
DMM: 1-deoxymannojirimycin
EDTA: ethylenediaminetetraacetic acid
Endo H: endoglycosidase H
ER: endoplasmic reticulum
FBS: fetal bovine serum
GA: Golgi apparatus
GFP: green fluorescent protein 130
GM130: Golgi matrix protein
HEK293: Human Embryonic Kidney 293 cells
HRP: horseradish peroxidase
M: membrane
NMDAR: *N*-methyl-D-aspartate receptors
PACSIN1: protein kinase C and casein kinase substrate in neurons 1
PFA: paraformaldehyde
PBS: phosphate-buffered saline
PDI: protein disulfide isomerase
PSD-95: postsynaptic density protein 95
PNGase F: peptide-*N*-glycosidase F
PVDF: polyvinylidene difluoride
P: postnatal day
QD: Quantum dot
SDS: sodium dodecyl sulfate
SDS-PAGE: sodium dodecyl sulfate-polyacrylamide gel electrophoresis
SEM: standard error of the mean
TX-100: Triton X-100.
