# Supplementary figures and images for: N-Glycosylation Regulates the Trafficking and Surface Mobility of GluN3A-Containing NMDA Receptors
Source: Front Mol Neurosci. 2018 Jun 4;11:188. doi: 10.3389/fnmol.2018.00188 (PMC5994540; doi:10.3389/fnmol.2018.00188)

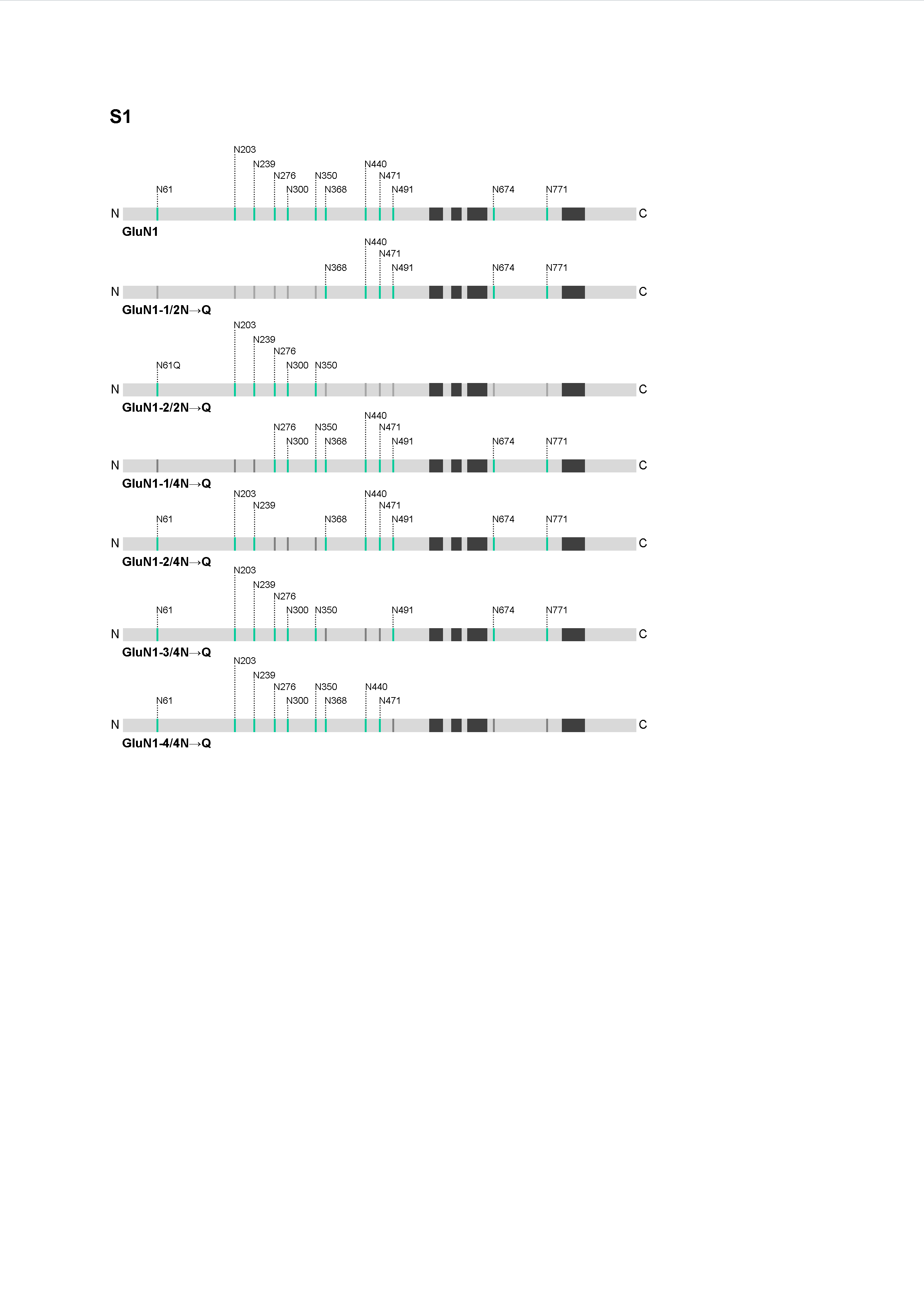

Supplement: Supplementary file 1 [file Image_1.TIF]

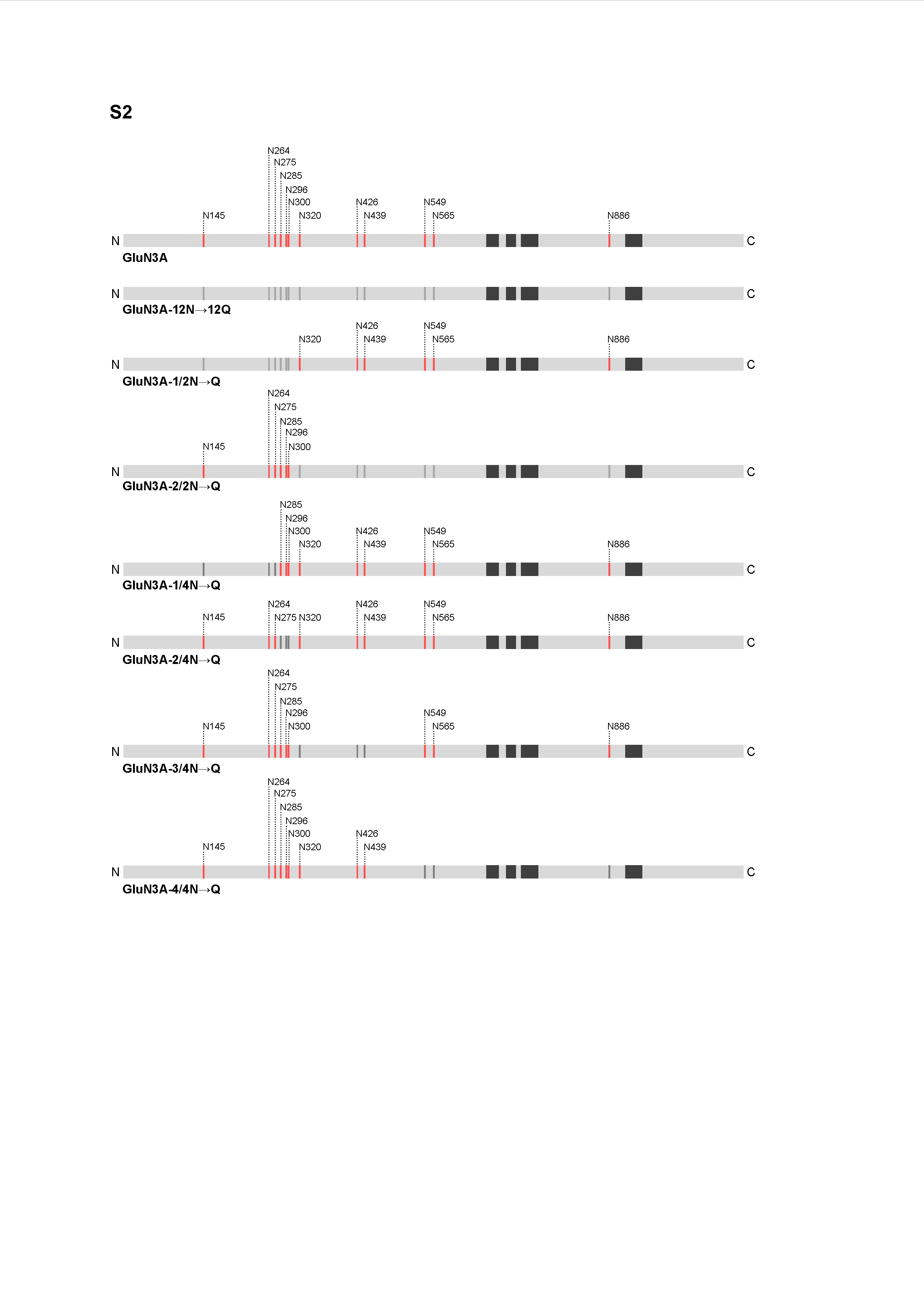

Supplement: Supplementary file 2 [file Image_2.TIF]
