# Supplementary material for: N-Glycosylation Regulates the Trafficking and Surface Mobility of GluN3A-Containing NMDA Receptors
Source: Front Mol Neurosci. 2018 Jun 4;11:188. doi: 10.3389/fnmol.2018.00188 (PMC5994540; doi:10.3389/fnmol.2018.00188)
Supplement: Supplementary file 3 [file Data_Sheet_1.pdf]

Supplementary Material

N-glycosylation regulates the trafficking and surface mobility of GluN3A-containing NMDA receptors

Kristyna Skrenkova[1,2,3#], Sanghyeon Lee[4,#], Katarina Lichnerova[1,2], Martina Kaniakova[2], Hana Hansikova[5], Martin Zapotocky[6], Young Ho Suh[4,*], Martin Horak[1,2,*]

*** Correspondence:**

Martin Horak, Department of Neurochemistry, Institute of Experimental Medicine of the Czech Academy of Sciences, Videnska 1083, 14220 Prague 4, Czech Republic; E-mail: martin.horak@iem.cas.cz

Young Ho Suh, Department of Biomedical Sciences, Seoul National University College of Medicine, 103 Daehak-ro, Jongno-gu, Seoul 03080, South Korea; E-mail: suhyho@snu.ac.kr

FIGURE LEGENDS

FIGURE S1. Schematic diagram of the 12 consensus *N*-glycosylation sites (N-X-S/T) in the GluN1 subunits. The generated mutant versions of the GluN1-4a subunit (GluN1) in which various combinations of the 12 consensus *N*-glycosylation sites were replaced with a glutamine residue (N→Q; disrupted *N*-glycosylation sites lack numbering and are shown in grey color). N=N-terminus; C=C-terminus; black rectangles indicate membrane domains. The following multiple-site mutant versions of the GluN1 constructs were generated: GluN1-N61Q-N203Q-N239Q- N276Q-N300Q-N350Q = GluN1-1/2N→Q; GluN1-N368Q-N440Q-N471Q-N491Q-N674Q-N771Q = GluN1-2/2N→Q; GluN1-N61Q-N203Q-N239Q = GluN1-1/4N→Q; GluN1-N276Q-N300Q-N350Q = GluN1-2/4N→Q; GluN1-N368Q-N440Q-N471Q = GluN1-3/4N→Q; GluN1-N491Q-N674Q-N771Q = GluN1-4/4N→Q.

FIGURE S2. Schematic diagram of the 12 consensus *N*-glycosylation sites (N-X-S/T) in the GluN3A subunits. The generated mutant versions of the GluN3A subunit in which various combinations of the 12 consensus *N*-glycosylation sites were replaced with a glutamine residue (N→Q; disrupted *N*-glycosylation sites lack numbering and are shown in grey color). N=N-terminus; C=C-terminus; black rectangles indicate membrane domains. The following multiple-site mutant versions of the GluN3A constructs were generated: GluN3A-N145Q-N264Q-N275Q-N285Q-N296Q-N300Q-N320Q-N426Q-N439Q-N549Q-N565Q-N886Q = GluN3A-12N→12Q; GluN3A-N145Q-N264Q-N275Q-N285Q-N296Q-N300Q = GluN3A-1/2N→Q; GluN3A-N320Q-N426Q-N439Q-N549Q-N565Q-N886Q = GluN3A-2/2N→Q GluN3A-N145Q-N264Q-N275Q = GluN3A-1/4N→Q; GluN3A-N285Q-N296Q-N300Q = GluN3A-2/4N→Q; GluN3A-N320Q-N426Q-N439Q = GluN3A-3/4N→Q; GluN3A-N549Q-N565Q-N886Q = GluN3A-4/4N→Q.
